# Risk of postoperative bleeding and thromboembolic events in anticoagulated patients undergoing transurethral resection of bladder tumors

**DOI:** 10.1177/17562872251315930

**Published:** 2025-02-04

**Authors:** Thomas Paul Scherer, Cici Dam, Uwe Bieri, Daniel Eberli, Raeto Strebel

**Affiliations:** Department of Urology, University Hospital Zurich, Frauenklinikstrasse 10, Zürich, CH 80091, Switzerland Department of Urology, Cantonal Hospital Graubuenden, Chur, Switzerland; Department of Urology, University Hospital Zurich, University of Zurich, Zurich, Switzerland; Department of Urology, University Hospital Zurich, University of Zurich, Zurich, Switzerland; Department of Urology, Cantonal Hospital Baden, Baden, Switzerland; Department of Urology, University Hospital Zurich, University of Zurich, Zurich, Switzerland; Department of Urology, Cantonal Hospital Graubuenden, Chur, Switzerland

**Keywords:** anticoagulation, atrial fibrillation, postoperative bleeding, thromboembolism

## Abstract

**Background:**

Transurethral resection of the bladder (TURB) harbors a high-risk for postoperative bleeding, especially in patients requiring anticoagulation. Recently, direct oral anticoagulants (DOACs) have become a popular alternative to vitamin K antagonists (VKAs), though their impact on TURB complications remains unclear.

**Objectives:**

To assess the postoperative complications of TURB from patients taking DOACs and VKAs.

**Design:**

Retrospective cohort study.

**Materials and methods:**

We retrospectively identified anticoagulated patients undergoing a TURB at our institution between 2012 and 2022 and divided them into two groups: whether they received VKA or DOAC. Follow-up of each patient was performed for 3 months. Occurrence and time to event of postoperative bleeding and thromboembolic events were recorded. A multivariable regression analysis was performed to assess risk differences.

**Results:**

A total of 167 patients (11.7%) fulfilled the inclusion criteria, of which 102 patients (61.1%) received a DOAC and 65 patients (38.9%) a VKA. Postoperative bleeding led to re-catheterization in 13 (12.8%) DOAC and 6 (9.2%) VKA patients (*p* = 0.49) and re-intervention in 7 (6.9%) DOAC and 4 (6.2%) VKA patients (*p* = 0.86). Blood transfusions were administered to 3 DOAC patients (2.9%), none in the VKA group. No thromboembolic events were reported.

**Conclusion:**

TURB carries low morbidity in anticoagulated patients. Thromboembolic events and the need for blood transfusion are infrequent. No substantial difference between the postoperative bleeding risk of patients receiving DOAC or VKA was found. All bleeding complications occurred within 2 weeks, marking it a potentially safe point in time to restart the OAC thereafter.

## Plain language summary

Risk of postoperative bleeding and thromboembolic events in anticoagulated patients undergoing transurethral resection of bladder tumors

**Background:**

Transurethral resection of the bladder (TURB) is a surgery with a high risk of bleeding after the operation, especially for patients who take blood-thinning medications (anticoagulants). Recently, newer anticoagulants called direct oral anticoagulants (DOACs) have become popular instead of the older vitamin K antagonists (VKAs), but it’s not clear how these newer drugs affect complications after TURB.

**Objectives:**

This study aims to compare the complications after TURB in patients taking either DOACs or VKAs.

**Study Design:**

Retrospective Analysis of patient data.

**Materials and Methods:**

The study reviewed records of patients on anticoagulants who had TURB at our institution between 2012 and 2022. Patients were divided into two groups based on whether they took VKAs or DOACs. Each patient was followed up for 3 months after the surgery. The researchers recorded how often and when bleeding and clotting events happened after surgery.

**Results:**

167 patients met the study criteria: 102 on DOACs and 65 on VKAs. Bleeding after surgery required re-catheterization in 13 patients (12.8%) on DOACs and 6 patients (9.2%) on VKAs, showing no significant difference. Re-intervention was needed for bleeding in 7 patients (6.9%) on DOACs and 4 patients (6.2%) on VKAs, also showing no significant difference. Blood transfusions were needed in 3 patients (2.9%) on DOACs and none on VKAs. No clotting events were reported.

**Conclusions:**

TURB is generally safe for patients on anticoagulants, with low rates of serious complications. There was no significant difference in the risk of postoperative bleeding between patients taking DOACs and those taking VKAs. All bleeding complications happened within 2 weeks after surgery, suggesting that it might be safe to restart blood thinners after this period.

## Introduction

Bladder cancer (BC), the ninth most common cancer globally, is more prevalent in men and significantly associated with increasing age and smoking.^1,2^ Substantial geographic differences exist, with 55% of BC cases and 43% of BC-associated deaths occurring in the 20% most developed countries. Furthermore, variations in the trends of age-standardized incidence rates have been observed across different regions. The incident rates in central, eastern, and southern Europe increased, while a decrease in central and eastern Asia as well as western and northern Europe was observed. The incidence in North America remained mostly stable.^3^ Tobacco smoking is by far the strongest modifiable risk factor for BC.^4^ Due to the increasing life-expectations global the absolute incidence of BC is expected to increase.^5^

Smoking and increasing age predispose patients not only to BC but also to multiple diseases which are complicating clinical management.^6,7^ Notably, 20% of all newly diagnosed patients with BC are found to have atrial fibrillation.^8^ Depending on their thromboembolic risk, patients with atrial fibrillation are anticoagulated to prevent thromboembolic events.^9^ Other reasons for anticoagulation include venous thromboembolism, mechanical heart valves, and thrombophilia.^10–12^

Historically, coumarins were the only available oral anticoagulation therapy (OAC). A coumarin acts as a competitive Vitamin K antagonist (VKA), lowering coagulation factors IX, X, II, VII.^13^ However, in the past decade, new oral anticoagulation drugs (DOAC) directly inhibiting factor IIa or Xa became more prevalent in the clinical routine.^13^ DOACs are administered in fixed doses, making them more convenient to use.^14^ Warfarin, which has a delayed onset and offset of action due to its indirect mechanism, requires bridging with a fast-acting parenteral anticoagulant at initiation. DOACs direct action allows them to reach peak levels within 1–4 h and provides a faster offset due to their shorter half-lives.^14^ Although randomized controlled trials have shown reduced bleeding complications of NOAC compared to warfarin, real-world data remain conflicting.^14^ In most indications, NOAC seems to be the preferred option of anticoagulation, but in patients with mechanical heart valves, VKA are still the recommended therapy.^15^

Transurethral resection of the bladder (TURB) represents the first step of the diagnostic pathway in BC and is classified as high-risk procedure for intra- and postoperative bleeding.^16–19^ The use of anticoagulation therapy further increases the risk of bleeding both during and after surgery.^20,21^ In the case of a severe hemorrhage, blood transfusions are required, carrying the potential not only for rare but also for potentially severe complications but are associated with increased morbidity and mortality in BC patients.^22–24^ Furthermore, a mechanical evacuation of the blood clot may be necessary due to urinary bladder tamponade, which poses the risk of a potential bladder wall rupture.^25^ Although guidelines for preoperative management of OAC exist, postoperative management is often individualized according to the patient’s risk.^26^ The literature on perioperative risk stratification and management in anticoagulated patients undergoing TURB is limited, with very few studies differentiating between antiplatelet and anticoagulation therapies.^21,27,28^ Hence, the exact risk of complications depending on the type of OAC in TURB is unknown. This study aimed to assess the outcomes related to thromboembolic events and postoperative bleeding among patients undergoing TURB, comparing those on DOACs with those on VKAs.

## Methods

In this retrospective cohort study, all patients who underwent transurethral resection of a bladder tumor between 11/2012 and 10/2022 at our tertiary care center were retrospectively reviewed for receiving oral anticoagulation therapy. The patient charts were manually reviewed between 02/2023 and 12/2023, and all outcomes were independently assessed by two authors (T.S. and C.D.). Patients with previous radiotherapy of the pelvis and mechanical aortic valves were excluded. We recorded whether the resection was mono- or bipolar, along with patient age, sex, BMI, date of operation, focality of the tumor, concomitant use of antiplatelet therapy (APT), T-stage, grade of BC, adjuvant treatment, comorbidities including preoperative CHA2DSVASC2, and preoperative HASBLED score, as well as the type of OAC, were recorded. All patients were retrospectively follow-up for 3 months postsurgery using the digital patient charts system.

Patients were categorized into the DOAC group or the VKA group based on the type of anticoagulation drug. The primary outcomes were postoperative bleeding, requiring transfusion (Clavien–Dindo II), re-catheterization (Clavien–Dindo IIIa), or re-intervention (Clavien–Dindo IIIb), and any venous thromboembolic events. Additionally, the pre- and postoperative management of the OAC therapy were recorded, specifically the cessation time of the anticoagulative drug, and if bridging with low molecular weight heparin (LMWH) or unfractionated heparin (UFH) has occurred.

Descriptive statistics were used for the baseline characters and differences were assessed using Pearson’s chi-squared test for binary variables and *t*-tests for continuous variables. To assess differences in postoperative bleeding complications, a multivariable logistic regression analysis was performed. The corresponding *p*-values were calculated with Wald tests. Predefined co-variables for the regression were age, sex, and continued use of APT and T Stage. Statistical significance was defined as a value of α < 0.05. To display the time distribution of the bleeding complications of our cohort a Lorenz Curve was plotted.

Data was recorded using Microsoft Access (Version 2016, Microsoft Corporation, Redmond, WA, USA). Statistical analysis was performed using STATA 18 (StataCorp., TX, USA). The internal data analysis and the follow-up of the patients were reviewed and approved by the local ethics committee (KEK Nr. 2022-02017). The reporting of this study conforms to the Strengthening the Reporting of Observational Studies in Epidemiology (STROBE) statement.^29^ The STROBE checklist Version 4 can be accessed in the Supplemental Files.

## Results

**Figure 1.**
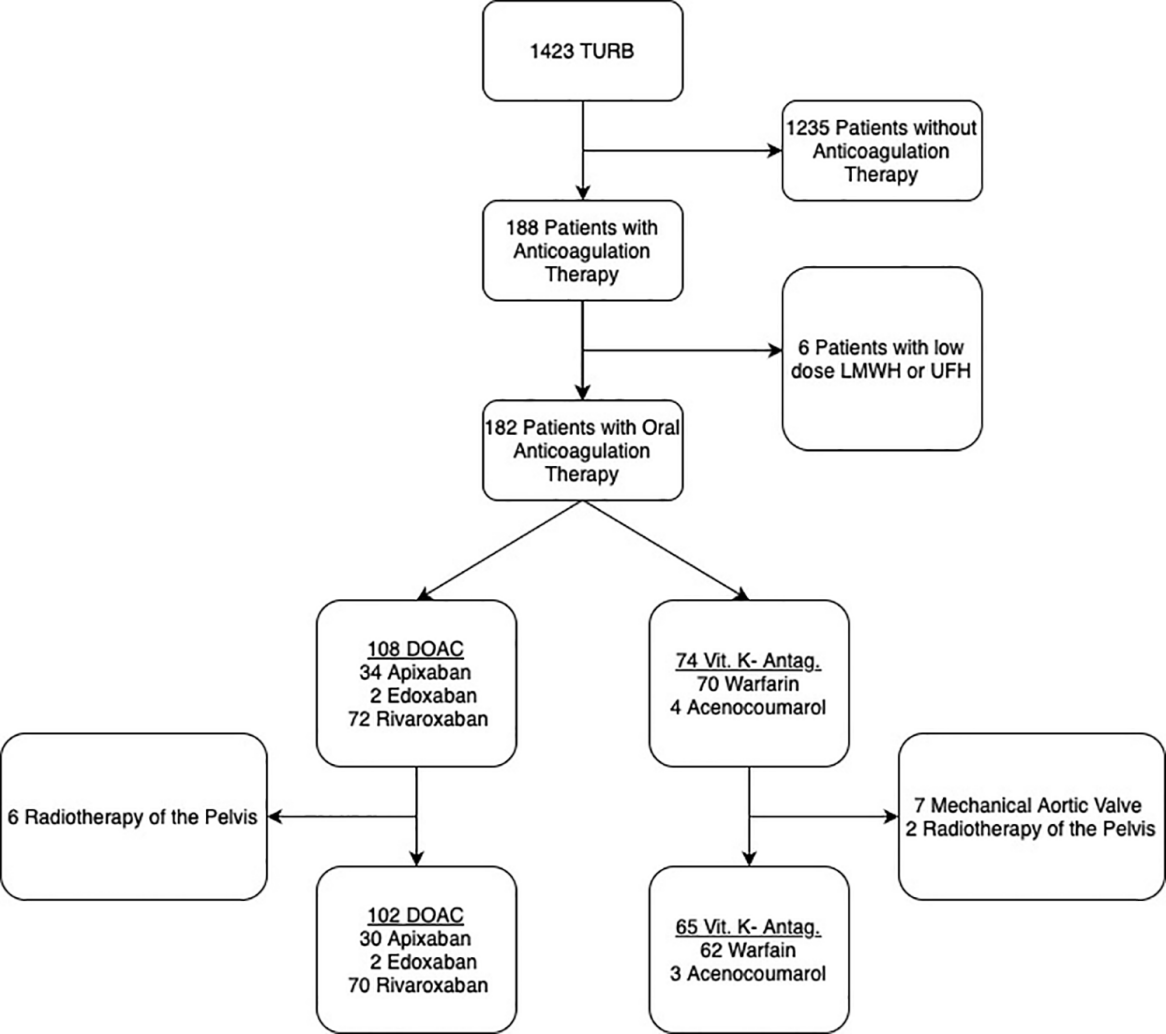
Study flow chart. DOAC, direct oral anticoagulants; LMWH, low molecular weight heparin; TURB, transurethral resection of bladder tumor; UFH, unfractionated heparin; Vit. K-Antag., vitamin K-antagonists.

Between 2012 and 2022, 1423 patients underwent a TURB at our institution (Figure 1). Among these, 167 patients (11.7%) fulfilled the inclusion criteria, of which 102 patients (61.1%) were anticoagulated with a DOAC and 65 patients (38.9%) with a VKA. All interventions were performed using bipolar resection. The baseline clinical characteristics of patients are illustrated in Table 1. The median age in the VKA group was 81 years (interquartile range (IQR): 78–87 years). Patients in the DOAC group had a median age of 78 years (IQR: 74–84 years). A complete 3-month follow-up was possible for all patients except one (1.5%) in the VKA group, who passed away 4 days postoperatively due to an aspiration pneumonia. The presence of concomitant APT was noted in 4 patients (6.2%) within the VKA group and in 21 patients (21.6%) in the DOAC group. Among these patients receiving concomitant APT, 2 patients (3.1%) in the VKA group and 17 patients (16.7%) in the DOAC group continued APT therapy perioperatively. The odds of patients in the VKA group presenting with emergency macrohematuria were twice as high compared to the DOAC group (16 (24.6%) vs 13 (12.8%)).

**Table 1.** Clinical characteristics of the patients eligible for the study. Renal insufficiency was defined as an estimated glomerular filtration rate of < 30 ml/min.

Patient characteristics	No. (%)		Total *n* = 167 (100%)	*p*-Values
	Vit. K antagonists *n* = 65 (38.9%)	DOAC *n* = 102 (61.1%)		
Age, years				
Median	81	78	80	0.010
IQR	78–87	74–84	75–85	
Sex, No. (%)				
Men	56 (86.2%)	92 (90.2%)	148 (88.6%)	0.42
Women	9 (13.9%)	10 9.8%)	19 (11.4%)	
Antiplatelet therapy, No. (%)				
ASA	3 (4.6%)	21 (20.6%)	24 (14.4%)	0.007
Clopidogrel	—	1 0.9%)	1 0.6%)	
Dual therapy	1 (1.5%)		1 0.6%)	
Comorbidities, No. (%)				
CHF history	24 (36.9%)	30 (29.4%)	54 (32.3%)	0.31
Hypertension	58 (89.2%)	84 (82.4%)	142 (85.0%)	0.22
Stroke or TIA history	28 (43.1%)	53 (52.0%)	81 (48.5%)	0.26
Vascular disease	23 (35.4%)	49 (48.0%)	72 (43.1%)	0.11
Diabetes	20 (30.8%)	27 (26.5%)	47 (28.1%)	0.55
Renal insufficiency	41 (63.1%)	63 (61.8%)	104 (62.3%)	0.86
Prior major bleeding	6 9.2%)	9 8.8%)	15 9.0%)	0.93
Alcohol use	14 (21.5%)	33 (32.4%)	47 (28.1%)	0.13
CHA2DSVASc2, No. (%)				0.20
1–3	16 (24.6%)	31 (30.4%)	47 (28.1%)	
4–5	30 (46.2%)	33 (32.4%)	63 (37.7%)	
⩾6	19 (29.2%)	38 (37.3%)	57 (34.1%)	
HASBLED, No. (%)				0.031
⩽2	13 (20.0%)	28 (27.5%)	41 (24.6%)	
3	35 (53.9%)	34 (33.3%)	69 (41.3%)	
⩾4	17 (26.2%)	40 (39.2%)	57 (34.1%)	
Intervention time, min				
Median	28	30	29	0.65
IQR	23–45	20–52	21–49	
Tumor focality, No. (%)				0.27
unifocal	23 (35.4%)	49 (48.0%)	72 (43.1%)	
multifocal	31 (47.7%)	39 (38.2%)	70 (41.9%)	
Unknown	11 (16.9%)	14 (13.7%)	25 (15.0%)	
Clinical T category, No. (%)				0.59
Benign	10 (15.4%)	14 (13.7%)	24 (14.4%)	
Ta	28 (43.1%)	55 (53.9%)	83 (49.7%)	
T1	10 (15.4%)	9 8.8%)	19 (11.4%)	
⩾T2a	15 (23.1%)	20 (19.6%)	35 (21.0%)	
Primary CIS	2 3.1%)	4 3.9%)	6 3.6%)	
Concomitant CIS	6 (14.8%)	5 (10.4%)	4 6.8%)	

ASA, acetylsalicylic acid; CIS, carcinoma in situ; CHF, congestive heart failure; DOAC, direct oral anticoagulants; min, minutes; No, number; TIA, transient ischemic attack; Vit. K, vitamin K.

The median hospital stay was 5.0 days (IQR: 3.1–6.9 days) for patients in the VKA group, compared to 3.1 days (IQR: 2.2–5.0 days) in the DOAC group. Notably, during the study period, the standard operating procedure for catheter removal after TURB was changed from 3 days to 2 days, reducing hospitalization times in both groups over time. For all scheduled procedures, the OAC was preoperatively paused. Patients in the VKA group discontinued their OAC for a median duration of 8.4 days (IQR: 5.4–11.4 days) prior to the intervention, with 91.8% underwent bridging procedure with LMWH or UFH. Bridging was not performed or could retrospectively not be verified in four patients (8.2%). Patients in the DOAC group were instructed to pause their OAC for a median of 2.4 days before the operation (IQR: 2.3–3.4 days), with only one patient undergoing bridging for unknown reasons with LMWH.

Following catheter removal, seven patients (10.8%) in the VKA group required postoperative re-catheterization. In the DOAC group, 20 patients (19.6%) underwent re-catheterization postoperatively. Among the patients requiring re-catheterization, bleeding was the cause in 6 (9.2%) cases in the VKA group and in 13 (12.8%) cases in the DOAC group. Operative reintervention due to macrohematuria was performed in four patients (6.2%) in the VKA group and seven patients (6.9%) in the DOAC group. Blood transfusions were administered to three patients (2.9%) in the DOAC group, who had a hemoglobin level below 7.0 g/dL. No blood transfusion was necessary in the VKA group. Of note, in both groups, no thromboembolic events were reported (Table 2).

**Table 2.** Complications after transurethral resection of bladder tumors.

Results	No. (%)			
	Vit. K Antagonists *n* = 65 (39%)	DOAC *n* = 102 (61%)	Total *n* = 167 (100%)	*p*-Values
Re-Catheterization, No. (%)				
Total	7 (10.8%)	20 (19.6%)	27 (16.2%)	0.13
Bleeding	6 (9.2%)	13 (12.8%)	19 (11.4%)	0.49
Urinary Retention	1 (1.5%)	7 (6.9%)	8 (4.8%)	0.12
Re-Intervention				
No. (%)	4 (6.2%)	7 (6.9%)	11 (6.6%)	0.86
Transfusion				
No. (%)	0 (0.0%)	3 (2.9%)	3 (1.8%)	0.16
Thromboembolic event				
No. (%)	0 (0.0%)	0 (0.0%)	0 (0.0%)	—

DOAC, direct oral anticoagulants; No, number; Vit. K, Vitamin K.

**Figure 2.**
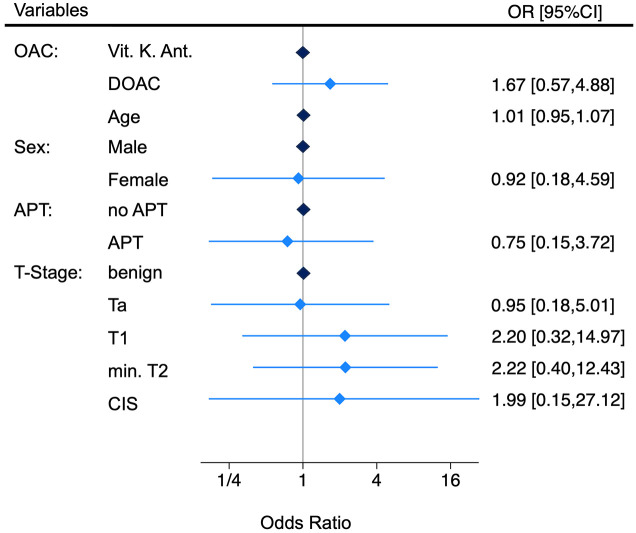
Odds ratios from the predefined logistic regression analysis for bleeding complications needing re-catheterizations. APT, continued antiplatelet therapy; DOAC, direct oral anticoagulants; OAC, oral anticoagulants; Vit. K-Ant., Vitamin K antagonists.

In the univariable logistic regression, which was unadjusted for other factors, the odds ratio (OR) for experiencing a bleeding event that required re-catheterization was 1.44 for patients receiving DOAC compared to the VKA group (95% confidence interval (95% CI) 0.52–3.99; *p* = 0.49). The results of the univariable logistic regression is displayed in Table 3. After adjusting for the pre-specified co-variables, the multivariable logistic regression resulted in an OR for experiencing a bleeding event that required re-catheterization of 1.67 (95% CI: 0.57–4.88; *p* = 0.35, Figure 2). In our analysis, correction for T stage and age had all only a moderate influence effect of the multivariable OR comparing the two drug classes, whereas sex and perioperatively continued APT had negligible effect. Due to limited events in some subgroups, we were unable to fully assess potential effect modification across all variable combinations.

**Table 3.** Results of the univariable logistic regression for experiencing a bleeding event that required re-catheterization.

Variables	OR	95% CI		*p*-Value
DOAC versus Vit. K Antagonist	1.44	0.52	3.99	0.49
Age	1.01	0.95	1.07	0.74
Women versus Men	0.91	0.19	4.27	0.90
APT versus no APT	0.91	0.19	4.27	0.90
T Stage: benign				
Ta	1.01	0.20	5.23	0.99
T1	2.06	0.31	13.81	0.46
⩾T2	2.28	0.42	12.38	0.34
CIS	2.20	0.17	29.31	0.55

95% CI, 95% confidence interval; APT, continued Antiplatelet therapy; DOAC, direct oral anticoagulants; OR, Odds Ratio; Vit. K, vitamin K.

**Figure 3.**
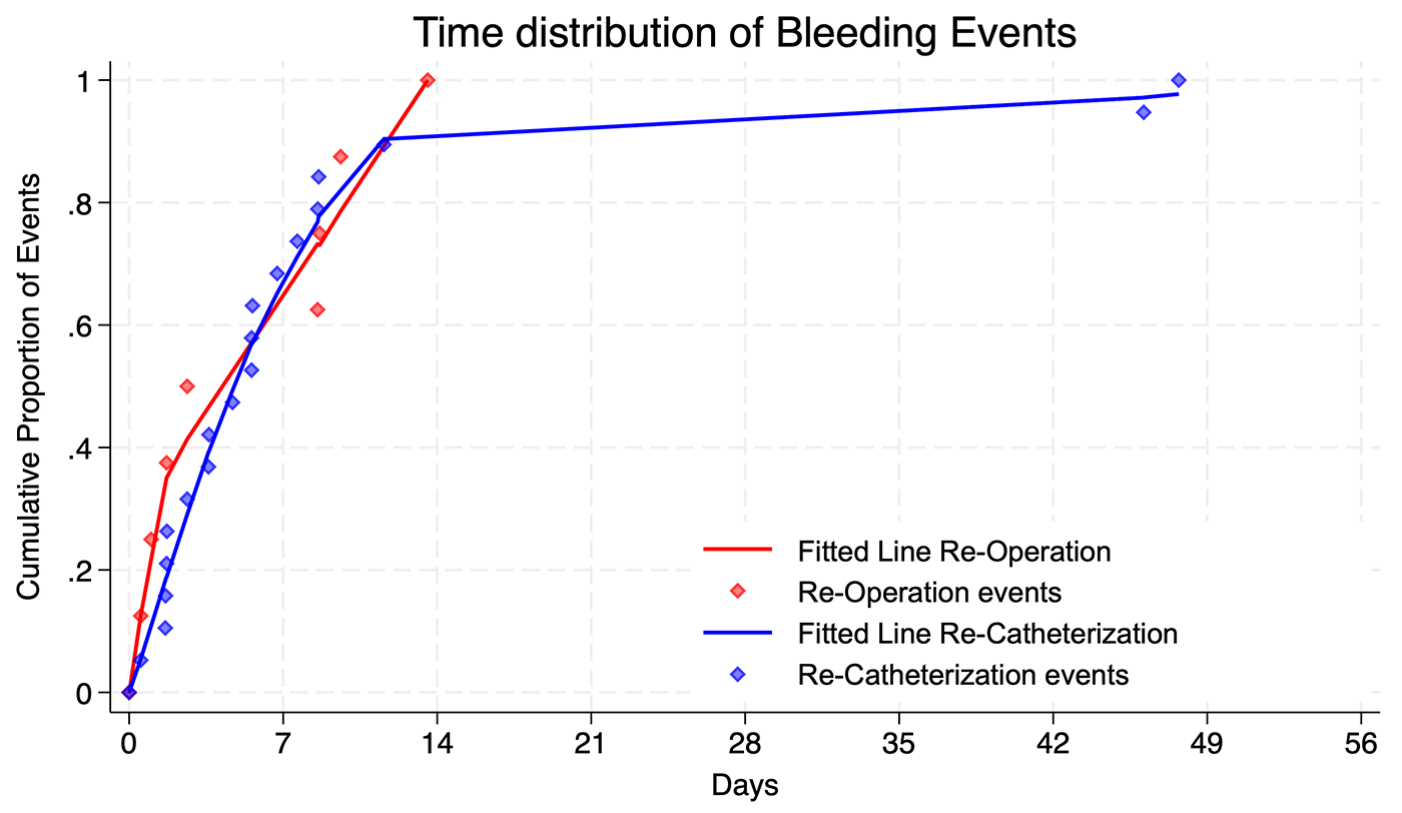
Lorenz curve illustrating the cumulative risk of bleeding complications across the entire TUR-B cohort. Red diamonds represent re-operation events, highlighted by a fitted red line, while blue diamonds represent re-catheterization events with a fitted blue line for visualization.

**Figure 4.**
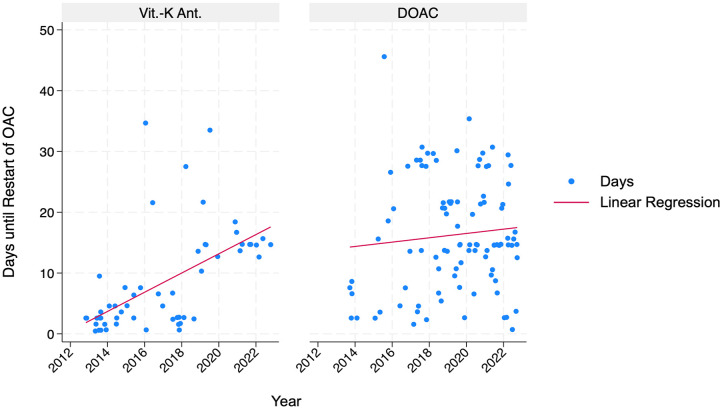
Scatter plot of time to resuming the OAC throughout the study period for the VKA and the DOAC group. DOAC, Direct Oral Anticoagulants; OAC, Oral Anticoagulants; Vit. K-Ant., Vitamin K Antagonists.

Regarding the onset of bleeding complications, 25% (95% CI: 11.9%–52.1%) of the events occurred within 24 h and 50% (95% CI: 43.5%–83.5%) happened within 6 days postoperatively. All re-operations due to bleeding were performed within 2 weeks (Figure 3). All patients were postoperatively started on thromboembolic prophylaxis low-dose LMWH or UFH depending on their kidney function. The duration of this postoperative bridging was significantly shorter in the VKA group with a mean of 8.4 days (95% CI: 6.2–10.6 days), compared to the DOAC group in which the OAC was reestablished after a mean of 16.3 days (95% CI: 14.4–18.2 days). However, this cessation time of OAC changed over time and became substantially longer in the VKA group in the observed period (Figure 4).

## Discussion

Our study presents a decade of data on anticoagulated patients receiving DOAC or VKA undergoing TURB at our tertiary care center. This study is one of a few that describes 90-day outcomes in TURB patients receiving different OAC and compares them to each other. Overall, a re-intervention rate under general anesthesia was necessary in 6.6% of the patients. Despite variations in the postoperative management regarding the resumption of the OAC, no thromboembolic event was observed among the 167 patients. While there was a trend toward more bleeding complications in the DOAC group, no statistical significant difference was found in the multivariable logistic regression analysis between patients receiving DOAC and VKA. Interestingly, during the observed period, which coincided with the broader adoption of DOAC, there was an observable delay before the resumption of the OAC, especially in the VKA group. The more cautious approach to resuming the OAC could reflect a subjective perception of an increased bleeding risk during the adoption of DOAC.

Literature on complications and management in anticoagulated patients undergoing TURB is limited. The European Association of Urology and the American Urological Association do not provide explicit recommendations in anticoagulated patients.^19,26^ Moreover, most studies on outcomes of anticoagulated patients do not distinguish between different types of anticoagulation and often combine the patients with APT with those on anticoagulants.^21,27,28^ But, optimizing the management of OACs is crucial to reduce patient morbidity. The perioperative period carries an elevated risk of thromboembolic events, which is potentially fatal.^30^ Equally important is the need to minimize post-operative bleeding, as such bleeding can necessitate re-catheterization for irrigation or even further intervention, leading to extended hospitalization with the associated risks and costs.

Hematuria is the most prevalent complication after TURB, affecting around 2%–10% of patients.^19,21,27,28^ De Nunzio et al. reported, in their prospective multicenter study, a re-intervention rate of 2.2% and a 10.5% prevalence of transient hematuria following TURB, both of which were lower than our presented results.^27^ However, it is essential to highlight that their cohort consisted of younger patients with lower stages of BC, and the 35% of anticoagulated patients in their cohort all discontinued their therapy a week before the intervention. Another large retrospective cohort reported hematuria in 7.9% of patients, leading to a readmission in 4.1% of cases. Within this cohort, anticoagulants or APT was associated with an increased OR of 1.46 (95% CI: 0.98–2.16) for any complication.^21^ Unfortunately, the majority of this study period predated the DOAC era, and the authors did not report the distribution of patients receiving APT and OAC. Konishi et al. distinguished in their retrospective study on TURB outcomes patients with OAC and APT as well as differentiated between patients who continued and discontinued the medication perioperatively. The patients who continued therapy were more likely to suffer from postoperative bleeding and clot retention. However, the study population consisted of 174 patients, of which only 14 received OAC, limiting the generalizability of their results.^28^ Ording compared the risk of spontaneous hematuria in patients with urologic cancer on DOACs versus VKAs and found no significant difference between the two drugs.^31^ This supports our results that both anticoagulants have similar safety profiles.

In our cohort, all patients received postoperative thromboembolic prophylaxis, with either subcutaneous LMWH or subcutaneous UFH, based on their kidney function, due to the short-acting times in case of potential postoperative bleeding. Notably, all observed postoperative bleeding complications needing a re-intervention under general anesthesia occurred within the first 2 weeks after surgery. Within this timeframe, over 90% of bleeding events needing a re-catheterization also occurred. Marking 2 weeks as a potential time point to restart patients safely on their regular OAC. It is worth highlighting that over 40% of the postoperative bleeding complications could be managed conservatively with irrigation only.

To our knowledge, no prior study has reported on incident times of bleeding complications after TURB in anticoagulated patients. In a systematic review of the risk of thromboembolic events and postoperative bleeding in general urological surgeries, Tikkinen et al. reported 90% of bleeding complications within 4 days postoperatively.^32^ This time model was based on a large randomized controlled trial that assessed the risk of patients undergoing noncardiac surgery and consisted of less than 17% “urologic or gynecologic” procedures.^33^ In a more recent study, the same research group analyzed a single arm of a prospective randomized controlled trial of non-cardiac surgeries and found that major bleeding complications occurred later. In the first 24 h in 42.7% of cases (95% CI: 40.9%–44.6%) and within 14 days postoperatively in 88.3% of cases (95% CI: 86.5%–90.2%).^34^ Unfortunately, the most recent, now discontinued, urological guidelines from the European Association of Urology on thromboprophylaxis reference major complications occurring within the short timeframe based on the systematic review.^26^

Our results demonstrate that TURB displays a prolonged postoperative bleeding risk of up to 2 weeks. Our multivariable regression model shows patients with higher T stages might have an elevated risk. This higher risk of bleeding seems intuitive due to an increased resection area and altered vessels in more aggressive, higher-stage tumors. Most of the patients on APT in our study continued their medication perioperatively and did not display an elevated risk of bleeding complications in the multivariable logistic regression analysis, which is in line with other studies in the field.^35,36^ The incidence of transfusions in our study was too rare to draw a robust conclusion between the two study groups. Altogether three patients (1.8%) required blood transfusions, all of whom were in the DOAC group. Notably, one patient presented himself as an emergent consultation due to hematuria and already exhibited preoperative anemia.

No thromboembolic event was reported in both study groups. Nonetheless, given the potential life threating nature of such a complication, every patient in our cohort received thromboembolic prophylaxis with LMWH or UFH immediately after surgery. However, strong evidence for postoperative bridging regimens does not exist. Retrospective studies implicate a possible increase in postoperative bleeding complications while not changing the risk of thromboembolic events.^37,38^ However, it should be emphasized that given the rare occurrence of thromboembolic events, the potential benefit of bridging is more challenging to prove. Furthermore, the absence of prospective studies makes the justification for discontinuing prophylaxis against a potentially life-threatening condition difficult.

In summary, our study demonstrates that pausing DOACs 48 h and VKAs 7 days with bridging prior to surgery yield comparable rates of post-operative macrohematuria requiring intervention. Additionally, the approach of bridging OACs with LMWH up to 2 weeks post-operatively and resuming them 2 weeks after surgery appears feasible, given the low incidence of thromboembolic events and postoperative bleeding in this period. Both DOACs and VKAs showed similar safety profiles when following these preoperative and postoperative management strategies. Prospective studies on the perioperative management of OAC patients are urgently needed. Especially valuable would be a randomized controlled study comparing various strategies for the postoperative management of OAC, including temporary discontinuation, prophylactic bridging and direct resumption of OAC, with a focus on risks for bleeding risk and thromboprophylaxis.

This study has some limitations. The retrospective design might have led to differences between the two analyzed groups. Additionally, the intraoperative description of tumor extent may have varied across surgeons and tumor characteristics like tumor extent could not be reliably analyzed. The postoperative management of the patient was based on individualized decisions and varied over time, both may have also influenced the incidence of postoperative complications. Another limitation is the relatively small sample size, which restricts the statistical power of the findings and may impact the reliability of subgroup analyses. Moreover, this study was conducted at a single center, which limits the external validity and generalizability of the results. Furthermore, due to the absence of systematic thromboembolic screening, only symptomatic thromboembolic events could have been detected, possibly leading to underestimating their actual incidence.

## Conclusion

Our study shows that a TURB carries low morbidity in anticoagulated patients, and APT can be safely continued perioperatively. Especially, thromboembolic events and the need for blood transfusion are very rare. A substantial difference between the postoperative bleeding risk of patients receiving DOAC or VKA does not seem to exist. All postoperative bleeding complications happened within 2 weeks, marking it a potential safe point in time to restart the OAC.

## Supplemental Material

sj-pdf-1-tau-10.1177_17562872251315930 – Supplemental material for Risk of postoperative bleeding and thromboembolic events in anticoagulated patients undergoing transurethral resection of bladder tumors Supplemental material, sj-pdf-1-tau-10.1177_17562872251315930 for Risk of postoperative bleeding and thromboembolic events in anticoagulated patients undergoing transurethral resection of bladder tumors by Thomas Paul Scherer, Cici Dam, Uwe Bieri, Daniel Eberli and Raeto Strebel in Therapeutic Advances in Urology

## Appendix

### Abbreviations

APT Antiplatelet therapy

ASA Acetylsalicylic acid

BC Bladder Cancer

BMI Body Mass Index

DOAC Direct Oral Anticoagulants

LMWH Low molecular weight heparin

NMIBC Non-muscle invasive bladder cancer

OAC Oral Anticoagulants

TURB Transurethral resection of the bladder

UFH Unfractionated heparin

VKA Vitamin K Antagonist

## Declarations

**Ethics approval and consent to participate:** The internal data analysis and the follow-up of the patients were reviewed and approved by the local ethics committee (approval number 2022-02017). According to the approval statement, all patients with a signed general consent for retrospective data analysis could be included in the study and the consent for data analysis for patients treated before the implementation of a general consent (March 2018) for anonymous data analysis at the institution was waived by the ethical committee. Local ethical committee: Cantonal Ethics Committee Zurich, Stampfenbachstrasse 121, 8090 Zürich, Switzerland. E-mail: info.kek@kek.zh.ch

**Consent for publication:** Not applicable.

**Availability of data and materials:** The data that support the findings of this study are available from the first author, TPS, upon reasonable request.
